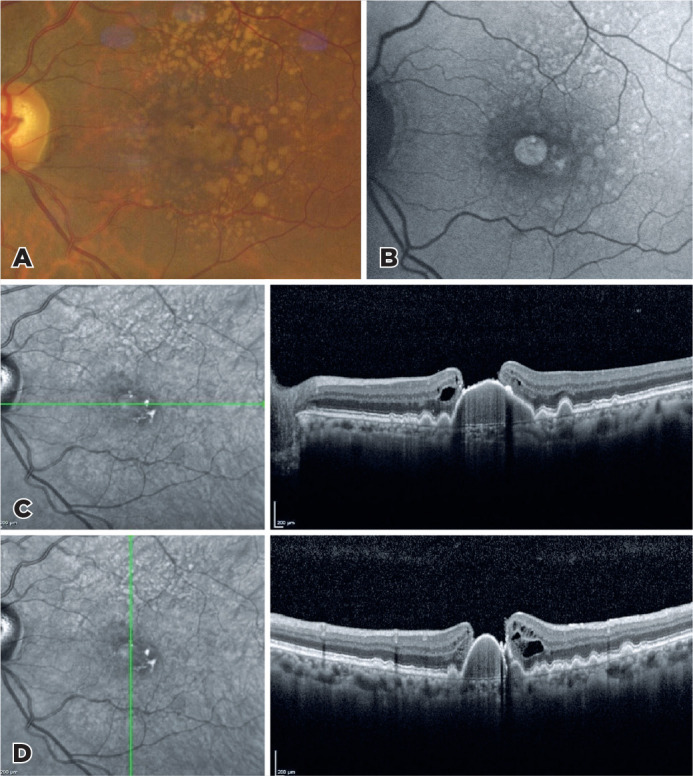# Full-thickness macular hole associated with drusenoid pigment epithelial detachment in age-related macular degeneration

**DOI:** 10.5935/0004-2749.2024-0292

**Published:** 2025-02-11

**Authors:** Kemal Tekin, Cemile Ucgul Atilgan

**Affiliations:** 1 Ophthalmology Department, Ulucanlar Eye Training and Research Hospital, Ankara, Turkey

A fundus image of a 70-year-old woman who has been followed up for age-related macular degeneration showed widespread soft drusen, aggregation of confluent drusen, and central macular hole with pigment epithelial detachment (A). The macular hole and drusen were hyperautofluorescence in fundus autofluorescence (B). Horizontal and vertical optical coherence tomography scans passing through the fovea showed subretinal pigment epithelium deposits, a large drusenoid pigment epithelial detachment combined with full-thickness macular hole^(1)^ with a detached posterior hyaloid (C,D).

**Figure:**